# The Fonthill Dental Surgery Complication Classification Scale

**DOI:** 10.1002/cre2.235

**Published:** 2019-08-11

**Authors:** Peter C. Fritz, Amanda B. Longo

**Affiliations:** ^1^ Dr. Peter C. Fritz Periodontal Wellness & Implant Surgery Fonthill ON Canada; ^2^ Department of Kinesiology, Faculty of Applied Health Sciences Brock University St. Catharines ON Canada; ^3^ Niagara Health Systems St. Catharines ON Canada

**Keywords:** classification, complication, dental, implant, periodontal

## Abstract

**Objectives:**

A lack of consensus on how to classify post‐operative complications in dentistry limits the ability for comparison of outcomes among treatments and their primary providers. Therefore, the Fonthill Dental Surgery Complication Classification Scale has been proposed as a uniform reporting tool to allow for the standardized quality assessment of dental treatment. This instrument classifies negative outcomes arising after dental treatment and is based on the clinician and the clinician time required to resolve the complication in seven classes of increasing severity.

**Materials and Methods:**

The scale was evaluated in a cohort of 2,382 consecutive patients, of which 9% experienced a complication, the majority of which were Class I or Class II—resolved without intervention by the dental surgeon.

**Results:**

Four scenarios where interpretation of the scale was required are presented with an explanation of their complication class.

**Conclusions:**

This classification system will ultimately prove reliable in measuring clinician success rate and aiding in the decision‐making process for patients, clinicians, and financial providers.

## 1 INTRODUCTION

It is said that every good surgeon remembers every negative outcome and there is a graveyard in every surgeon's mind where the complications rest. Despite best intentions, proper treatment planning, and effort, there is an inherent risk for complications to arise from every medical and dental procedure performed.

Nearly 30 years ago, a paradigm shift in the identification and measurement of medical complications was introduced with the development of a standardized reporting system of negative outcomes (Clavien, Sanabria, & Strasberg, 1992). Presently, there is no consensus on how to evaluate and report negative outcomes in the field of dentistry. Therefore, this seminal report aims to propose a new system specific to dentistry, the Fonthill Dental Surgery Complication Classification Scale, a novel grading system for classifying surgical complications in dental practice based on seven classes of increasing severity.

The current lack of standardized reporting of negative outcomes in dentistry creates an inability to accurately compare outcome data across the dental literature and to perform meta‐analyses. Similarly, nonuniform reporting also limits the ability for patients and financial providers to objectively compare treatment centers, individual clinicians, clinicians working in a team approach, and the success rate of different clinical techniques. As such, the lack of standardized comparative date following dental surgery is a major obstacle in providing the most cost‐effective and highest quality patient care.

Innovations in surgical and nonsurgical techniques have triggered a demand for a standardized method to assess their efficacy and the efficacy of the clinician (whether specialist or generalist). Due to the rising cost of dental care, objective and reliable outcome data are increasingly requested by patients and financial providers (private insurance or government funded). Therefore, the proposal of a standardized, global, easy‐to‐use dental complication reporting system represents a powerful opportunity to limit the costs of health care and empower patients and financial providers to statistically manage risk. Such complication classification scales exist in the medical discipline (Dindo, Demartines, & Clavien, 2004; Strasber, Linehan, & Hawkins, 2009) and are now widely used as compelling instruments for quality assessment, applicable worldwide and across a wide range of surgical specialties (Clavien et al., 2009).

Some literature involving the classification, etiology, and management of dental complications exists (Annibali, Ripari, La Monaca, Tonolo, & Cristalli, 2009; Boynes, Moore, Lewis, Zovko, & Close, 2010; Fontana, Maschera, Rocchietta, & Simion, 2011; Li & Wang, 2008; Park & Wang, 2005). However, the focus of these publications mainly involves technique‐related complications and their management. Descriptions of how to classify and categorize these postoperative outcomes in a standardized way are not included in these previously published studies. Therefore, the objective of this report is to (a) propose a scale modelled after the globally recognized Clavien–Dindo classification of surgical complications (Clavien et al., 2009) and (b) to validate this scale in a periodontal specialty clinic. The newly proposed scale for classifying complications in dental practice is based on seven classes of increasing severity. The proposed model was also tested in a large cohort of patients who underwent periodontal therapy in a private periodontal surgical specialty clinic over a 6‐month period.

## 2 CLINICAL INNOVATION REPORT

In order to identify complications, they must first be differentiated from other negative outcomes. Similar to the medical field, negative outcomes can be separated into three categories: complications, sequelae, and failure to cure (Clavien et al., 1992). A complication has been defined as any deviation from the normal postoperative course (Annibali et al., 2009) and require some level of treatment to resolve. Sequelae is a direct aftereffect of any procedure that is inherent to the procedure itself (Clavien et al., 1992). For example, after the extraction of a tooth, there is an inability (or reduced ability) for mastication. Lastly, although a procedure may be well executed, it may still fail. Although still a negative outcome, if the original purpose of the procedure has not been achieved, this is a failure to cure.

The Fonthill Dental Surgery Complication Classification Scale is presented in Table 1. This scale consists of seven classes, each increasing in severity and based on the time and the dental personnel required to fully resolve the complication arising from the initial dental treatment. Class I and Class II complications are resolved with dental support personnel only (i.e., dental office administrative staff, RDH, or those dental personnel practicing under the order of the dental surgeon) and without the intervention by the dental surgeon. Class III and Class IV complications require intervention by the dental surgeon. Class V complications require additional intervention by the dental surgeon and the expertise of another dental specialist. Class VI complications require intervention by a medical professional outside the field of dentistry for full resolution of the complication.

**Table 1:** Fonthill Dental Complication Classification Scale

Class	Definition
1	Any deviation from the normal postoperative course without the need for pharmacological treatment, dental, or surgical intervention.
	Full resolution can be obtained over the phone/text/email by dental personnel or by the dental surgeon.
2	Any deviation from the normal postoperative course without the need for intervention by the dental surgeon.
	The patient may need to return to the dental clinic but does not need to see the dental surgeon for full resolution of the complication.
3	Any deviation from the normal postoperative course requiring surgical, dental, or prosthetic intervention by the dental surgeon.
	The patient and dental surgeon need to meet for full resolution of the complication.
	Full resolution of the complication requires exactly one appointment.
4	Any deviation from the normal postoperative course requiring surgical, dental, or prosthetic intervention by the dental surgeon.
	The patient and dental surgeon need to meet for full resolution of the complication.
	Full resolution of the complication requires two or more appointments.
5	Any deviation from the normal postoperative course requiring surgical, dental, or prosthetic intervention by the dental surgeon and a third‐party dental specialist.
	Full resolution of the complication requires one or more appointments with the dental surgeon and one or more appointments with a third‐party dental specialist.
6	Any deviation from the normal postoperative course requiring intervention outside of the scope of dentistry (i.e., requiring medical intervention).
	Full resolution of the complication is beyond the scope of dentistry.
7	Death of a patient.

### 2.1 Validation in a cohort of 2,382 patients undergoing periodontal therapy

All patients having undergone periodontal surgery (i.e., biopsy, extraction, frenectomy, dental implant surgery, gingival grafting, periodontal flap surgery, and other) or nonsurgical periodontal therapy (i.e., scaling and root planing and supportive periodontal therapy) over a 6‐month period (January–June 2018) were tracked. Other procedures include buccal and lingual frenectomy, distal wedge, exposure of unerupted teeth, gingivectomy, gingivoplasty, ligament separation, and vestibular depth recontouring. All complications were classified retrospectively according to the Fonthill Dental Surgery Complication Classification Scale in a private periodontal clinic (Fonthill, ON). All periodontal surgery was performed by a single periodontist. All nonsurgical periodontal therapy was performed by one of five registered dental hygienists under the direct order of the periodontist. Complications were identified and classified from the chart notes of each patient that were created at each patient interaction through a digital, standardized template. Complications were counted and categorized by one of two trained and calibrated research associates.

The cohort retrospectively analyzed was made up of 2,382 patients. A complication was recorded in 9.02% of patients in the cohort (Table 2). Of all complications recorded, Class I complications accounted for 3.69% of the total, Class II for 2.43%, Class III for 1.81%, Class IV for 0.38%, Class V for 0.63%, and Class VI for 0.08%. There were no Class VII complications reported.

**Table 2:** Complications from a cohort of patients undergoing periodontal therapy

Complication rate (%)								
Therapy	1	2	3	4	5	6	7	Total
Biopsy	8.70	0.00	4.35	0.00	2.17	0.00	0.00	15.22
Extraction	5.19	3.70	5.19	0.00	0.00	0.00	0.00	14.08
Flap	2.33	6.98	4.65	0.00	0.00	0.00	0.00	13.96
Graft	18.06	11.81	5.56	0.69	0.00	0.00	0.00	36.12
Implant	8.98	6.89	7.19	2.40	2.40	0.60	0.00	28.46
Other Sx	8.11	8.11	0.00	0.00	0.00	0.00	0.00	16.22
Nonsurgical	0.63	0.42	0.00	0.00	0.42	0.00	0.00	1.47
Total	3.69	2.43	1.81	0.38	0.63	0.08	0.00	9.02

Of the complications reported, the most common were identified as Class I, Class II, or Class III. The two most common complications reported involved postoperative clarification of instructions and expectations. Common examples of this broad category of Class I complications included questions from patients such as “Can I remove my stent?,” “Are the symptoms (i.e., swelling, bleeding, pain, etc.) I am experiencing a part of normal healing?,” and “When can I begin to exercise?.” Common examples of each of the complication classes are outlined in Table 3. Full resolution of these Class I complications was accomplished by dental support personnel, without the need for the dental surgeon as many of these examples surround the patient's expectations around the magnitude of known complications to the surgery, such as swelling, bleeding, and postoperative care. A Class I complication occurred in 18% of all patients undergoing gingival grafting surgery.

**Table 3:** Examples of the Fonthill Dental Complication Classification Scale

Class	Definition
1	a. Patient requesting further postoperative instructions (i.e., diet modifications, activity modifications, and oral hygiene modifications) b. Patient requesting clarity of postoperative expectations (i.e., bleeding, swelling, pain, and bruising)
2	a. Patient requesting suture trimming b. Patient requesting polish from chlorhexidine staining c. Patient requesting a check of the healing site by supportive dental personnel
3	a. Patient requesting a check of a healing site, unresolved by dental support personnel b. Loose/lost healing abutment c. Patient requiring a new or different prescription (i.e., antibiotic, chlorhexidine rinse, and pain medication)
4	a. Failed surgery (i.e., failed implant and necrotic graft)
5	a. Lost or damaged crown or filling b. Neighboring tooth requiring a root canal as a result of complication during surgery
6	a. Aspirated instruments b. Allergic reaction to prescribed antibiotic requiring hospitalization c. Cardiac arrest during procedure d. Poor response to sedation requiring hospitalization
7	a. Death of a patient

The third and fourth most common complications reported were Class II and involved the need for the patient to return to the dental clinic for full resolution of the problem, but without the need to see the dental surgeon. The two most commonly reported Class II complications were long, hanging sutures causing irritation needing to be trimmed and chlorhexidine staining needing to be removed. Again, Class II complications were most likely reported in patients following gingival grafting surgery, with 12% of patients reporting a Class II complication.

Class III complications involving the need for the patient to meet with the dental surgeon for exactly one appointment occurred in only 1.8% of all patients and in 7% of dental implant surgery cases over this 6‐month period. The most common reason for this complication resolution was due to a lost or loose healing abutment after dental implant surgery. Postoperative pain that could not be resolved first by Class I or Class II intervention was also a common Class III complication reported.

Class IV complications were rare and occurred in only 0.38% of all patients (2.4% of dental implant cases). This suggests an implant success rate of nearly 98% of all patients. Interestingly, Class V complications occurred in 0.4% of patients undergoing nonsurgical periodontal therapy. Class V complications require intervention from a third‐party dental specialist, likely a generalist needing to replace or repair a restoration dislodged as a result of scaling and root planing by the RDH. Similarly, Class VI complications were very low, occurring in only 0.08% of all patients. Class VI complications were due to referring a patient for bloodwork postoperatively to identify a blood‐clotting disorder and a patient requiring emergency medical attention for an allergic reaction. These complications required expertise outside of the field of dentistry for their full resolution. There were no reports of a Class VII complication.

### 2.2 Evaluation and interpretation of indirect negative outcome resolution

As with the proposal of any reporting scale, there are instances in which the proposed system for classification is not clear and requires some interpretation. Below, we depict a number of scenarios in which the complication grade is not intuitively clear.

#### 2.2.1 Scenario 1: Patients developing complications of increasing severity following one surgical intervention

This scenario describes cases with the severity of the complication increasing over time. For example, a patient may call with a complaint of postoperative pain that at first can be resolved with clearer postoperative instructions from the dental personnel (Class I). This may escalate to the need to visit the clinic for assessment (Class II) and even further to see the dental surgeon one or more times for full resolution (Class III and/or Class IV). The difficulty lies in whether each grade of the complication must be recorded as individual complications or only the most severe. Only the most severe complication should be recorded for the most accurate record keeping and assessment of a treatment provider.

#### 2.2.2 Scenario 2: Referral from another surgeon following a surgical procedure with resulting complications

Commonly, specialists are referred to cases following complications caused by another dental surgeon. For example, placement of dental implants can sometimes result in peri‐implantitis requiring periodontal flap surgery or removal of the implant by a dental specialist (i.e., periodontist) for its resolution (Class V). When this complication is inevitably transferred, the dental specialist may be tempted not to include the initial surgery as a complication, but for quality recording, this should be considered a Class V complication with an addendum of “referred patient” to indicate that the source of the complication was from another dental surgeon.

#### 2.2.3 Scenario 3: Complication still present after follow‐up

This example depicts a complication that is not yet resolved after a first attempt at its resolution. For example, a patient may require more than one call to the pharmacy to ensure the correct dosage of medication for their specific course of recovery. Although this depicts two Class III complications, the case should only be classified as one Class III complication. However, should the patient require two visits with the dental surgeon, this escalates a complication from Class III to Class IV. Similar to Scenario 2, only the single most severe complication should be reported.

#### 2.2.4 Scenario 4: Dental surgeon retains patients when others may refer them to another dental or medical specialist

The treatment‐based model of classification of complications relies on the assumption that all dental surgeons take a team‐based approach. However, some retain patients who have experienced a negative outcome in order to resolve the problem themselves. In doing so, this will not escalate the complication class appropriately (i.e., Class IV to Class V or Class V to Class VI). In these instances, as long as full resolution of the negative outcome is achieved, whether the patient is retained or referred to another specialist is appropriate. However, clinicians must determine if this is the most cost‐effective and the best possible solution for the patient, all factors considered.

## 3 DISCUSSION

This article proposes a framework for classifying and reporting dental complications ranging in severity. Findings from a cohort of 2,382 patients undergoing periodontal therapy show 9.02% of patients experienced a complication, with the most common complications being of Class I.

The current landscape of dentistry does not encourage open discussion and reporting of complications experienced by patients. Governing bodies require the disclosure of malpractice and professional negligence, but the tracking of complications to progress the field of dentistry, periodontics, and implant surgery by allowing for comparisons is not a common practice.

Having a framework for increased transparency and creating a common language to openly discuss complications will also reduce underreporting of complications as this is an impartial and inclusive instrument that ultimately evaluates performance and leads to perpetual improvement.

With the institution of this scale, we propose its use as a determinant for measuring a clinician's success rate. Specifically, the Class IV complication rate for dental implant placement can be calculated and is directly reflective of the implant failure rate. In the cohort of patients analyzed in this study, only 2.4% experienced a Class IV complication, suggesting that the surgeon has an implant success rate of 97.6%. This metric will aid in the decision‐making process for patients and we suspect will one day influence the regulation and directives of financial providers.

Furthermore, reliable outcome data are becoming more crucial due to the rising cost of health care. From the patient, to the clinician, to the payer, all are uniquely aware of the cost of dental care, and reliable performance data should be made available when identifying the “best” surgeon for their dental needs. However, the current lack of a standardized scale for reporting complications, as well as the risk for sanctions by governing bodies and malpractice, claims frightens clinicians, and, as such, complications are often underreported and progress is inhibited to the disadvantage of all of those involved.

The major emphasis of the Fonthill Dental Surgery Complication Classification Scale uses the dental personnel time required to fully resolve the complication, a factor that does not vary significantly across the globe. Although postoperative complications are poorly documented, the treatment required is generally well reported in patient charts, which facilitates retrospective analysis. This approach reduces subjective interpretation and narrows the room for up‐ or down‐grading complications because it is based on clear parameters that are universally applicable, the surgeon's time. From the dental perspective, the clinician's time is considered the critical resource and is the most universally applicable metric to measure the severity of a complication. The patient perspective is also of great importance. However, there is great variance in the patient perspective after dental surgery depending on the patient's past experience, their current expectations, and the condition of the patient before and after dental surgery. Therefore, for simplicity and reliability, patient reported outcome measures were not included in this classification system.

Limitations to this scale exist, including the discrepancies that can arise in cases that are not straightforward. From a cohort of 2,382 patients from a private periodontal surgical center in Fonthill, Ontario, some of these discrepancies were noted and a recommendation for how to interpret the scale in those scenarios is outlined with clear reasoning. It can also be argued that the management of a specific complication will differ among clinicians or centers. This discrepancy in treatment may be due to differing clinical opinions or may depend on the ability and resources available to the surgeon themselves. As an example, some dental office administrators are equipped with knowledge and training to solve Class I complications, which, in the hands of the less experienced, may be escalated to Class II or Class III. The proposed scale also does not take time into account. Purposefully, no limit to the amount of time elapsed between the original dental procedure, and the resulting negative outcome was defined, as complications may take time to develop.

Lastly, subjectivity is a potential limitation in the use of such a proposed scale. To limit any confusion, clear and well‐defined verbiage was used to omit any confusion and to limit errors in reporting. To explore the potential limitation of subjectivity and to ascertain the reproducibility of this classification system, a global validation of the scale must be completed. Global validation by dental surgeons practicing around the world will appraise the Fonthill Dental Surgery Complication Classification Scale against different training and practice methodologies, ultimately testing the applicability and clarity as a measuring tool.

The proposed scale based on the clinician's time required to fully resolve the postoperative complication introduces a predictable framework for the field of dentistry to use in the assessment and comparison of outcomes among different techniques, clinicians, and treatment centers. This classification system will ultimately ensure patient safety and improve surgical innovation and best practices for all stakeholders.

## CONFLICT OF INTEREST

P. C. F. and A. B. L. manage a boutique, global consulting firm for periodontists, Cloak & Scalpel. The authors have no conflicts of interest to declare.

## FUNDING INFORMATION

No funding was granted for this Clinical Innovation Report.

## CLINICAL RELEVANCE

Scientific rationale for the study: There is no consensus on how to classify postoperative complications in dentistry, limiting the ability to compare therapies and providers when patients, financial providers, and clinicians are determining best practices.

Principle findings: In a cohort of 2,382 consecutive patients, 9% experienced a complication. Of all complications, 3.7%, 2.4%, and 1.8% of complications were Class I, Class II, and Class III, respectively. The remainder of complications were of Class IV and Class V. The Class IV complication rate for implant placement was 2.4%.

Practical implications: A seven‐class scale based primarily on the clinician and the clinician's time required to fully resolve the complication has been proposed as a standardized method or classifying negative outcomes postoperatively.
